# Higher Light Intensity Combined with Optimized Photoperiod Enhances Growth and Tassel Development in Maize Inbred Line

**DOI:** 10.3390/plants15081208

**Published:** 2026-04-15

**Authors:** Xiang Ji, Luming Zhong, Jun Liu, Qing Zhou, Dongxian He

**Affiliations:** Key Laboratory of Agricultural Engineering in Structure and Environment of MOARA, College of Water Resources & Civil Engineering, China Agricultural University, Beijing 100083, China; jx@cau.edu.cn (X.J.); luming@cau.edu.cn (L.Z.); jun.liu@cau.edu.cn (J.L.); cauzhou@cau.edu.cn (Q.Z.)

**Keywords:** maize, LED lighting, daily light integral (DLI), speed breeding, tassel development

## Abstract

Maize has a long generation cycle and sensitivity to photoperiod, which limit breeding efficiency. An LED plant factory with suitable light conditions provides a promising approach to overcoming challenges in speed breeding. This study optimized the LED light environment to enhance growth and tassel development in the maize inbred line from the V3 to V9 stages. Six lighting treatments were tested, combining three light intensities (800, 1200, and 1600 μmol m^−2^ s^−1^) and two photoperiods (10 h d^−1^ and 12 h d^−1^). Treatment with a light intensity of 1600 μmol m^−2^ s^−1^ and a photoperiod of 10 h d^−1^ resulted in the highest shoot fresh weight (396.9 g per plant), shoot dry weight (42.4 g per plant), leaf area (51.3 dm^2^ per plant), and stomatal length (34.6 μm), as well as improved photosystem performance. Furthermore, this treatment promoted tassel development, with the tassel length at the V9 stage being 45.8% longer than that under the treatment with a light intensity of 800 μmol m^−2^ s^−1^ and a photoperiod of 10 h d^−1^. These findings establish an optimized lighting strategy that significantly enhances the growth and tassel development of maize inbred lines from the V3 to V9 stages, providing a suitable light environment for maize speed breeding in plant factory systems.

## 1. Introduction

Maize (*Zea mays* L.) is one of the most widely cultivated C_4_ cereal crops globally and serves a crucial function in ensuring food security around the world. Traditional maize breeding conducted under field conditions is restricted by seasonal cycles, limiting generation turnover to one or two per year [1]. The development of a new cultivar often takes close to ten years, which constrains the ability of breeding programs to respond efficiently to evolving market demands and climate change [2]. An LED plant factory with optimized light conditions provides a promising approach to overcoming challenges in maize breeding.

Speed breeding, originally demonstrated in spring wheat and barley using extended photoperiods to achieve up to 4–6 generations per year [3,4], has emerged as a transformative approach for accelerating crop development. Subsequent studies have extended the application of speed breeding strategies to other crops. In long-day species, extending the photoperiod has proven highly effective for accelerating breeding cycles. For example, winter wheat cultivars subjected to optimized vernalization combined with a 22 h d^−1^ photoperiod completed a generation in approximately 87 days, enabling up to four generations per year [5]. Similarly, alfalfa grown under full-spectrum LED lighting with moderate light intensity and extended daylength reached flowering within 37 days across cultivars with contrasting dormancy characteristics [6]. In contrast, speed breeding strategies for short-day crops require the species-specific optimization of photoperiod, light intensity, and spectral composition to prevent reproductive inhibition. By incorporating optimized LED light spectra with embryo rescue, breeding cycles in cotton were shortened to 71–85 days, facilitating the possibility of producing as many as five generations annually [7]. Rice completed a full generation within 63 days under precisely regulated LED conditions, permitting 5–6 generations annually [8]. In soybean, red and white LED lighting combined with an 8 h photoperiod significantly accelerated flowering and maturity while maintaining seed viability [9]. Hemp, a highly photoperiod-sensitive crop, achieved a seed-to-seed cycle of only 61 days through sequential continuous-light and short-day treatments [10]. These studies demonstrate that although speed breeding has been successfully applied across diverse crop types, its effectiveness depends on species-specific coordination of light intensity, photoperiod, and cultivation systems, underscoring the necessity of tailored lighting strategies when extending speed breeding approaches to additional crops [11,12,13,14].

Maize was domesticated from the short-day species teosinte (*Zea mays* ssp., *parviglumis*) and retains characteristics of its short-day origin; thus, excessively long photoperiods may delay flowering [15,16]. Therefore, optimizing light intensity and photoperiod is crucial for promoting growth and accelerated flowering in maize speed breeding protocol [17]. Maize flowering is regulated by an integrated photoperiod–circadian network, in which *GIGANTEA* homologs (*ZmGI1* and *ZmGI2*) play central roles. *ZmGI1* loss-of-function accelerates juvenile-to-adult phase transition and flowering under long days [18], while *ZmGI2* modulates flowering through multiple interconnected pathways involving *ZmZCN8* and *ZmFPF1* [19,20,21].

Light intensity and photoperiod are two major environmental factors regulating maize growth, photosynthetic performance, and reproductive development. Previous studies have shown that insufficient light reduces photosynthesis, dry matter accumulation, nitrogen metabolism, and grain filling, whereas prolonged daylength may delay flowering and alter plant architecture and inflorescence development in maize [15,22,23]. Despite extensive research across crops, a systematic understanding of how combined light intensity and photoperiod affect maize morphology, tassel development, photosynthetic performance and photosystem II (PSII) activity in plant factory systems remains lacking. Because maize exhibits photoperiod sensitivity and a highly efficient C_4_ photosynthetic system, optimization of light intensity and photoperiod is critical for accelerating maize speed breeding.

Therefore, this study investigated the combined effects of light intensity and photoperiod treatment on morphological traits, tassel development, photosynthetic performance, and chlorophyll fluorescence in a maize inbred line during the critical floral initiation period, from the V3 to V9 stages, under plant factory conditions [15]. By integrating morphological, physiological, and photochemical indicators, this work aims to identify a light regime that maximizes photosynthesis while preventing delayed flowering associated with a long photoperiod, thereby providing a physiological foundation for maize speed breeding.

## 2. Results

### 2.1. Morphology of Maize Inbred Line

**Figure 1:**
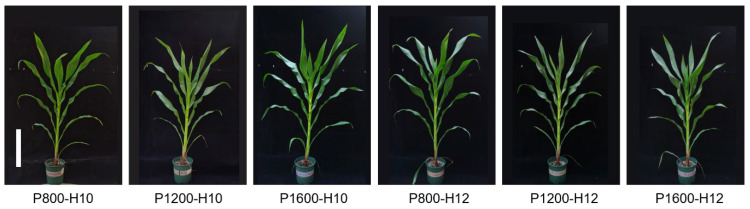
Representative photographs of maize inbred line (cv. Chang 7-2) in response to different light intensities and photoperiods at V9 stage. Scale bar = 50 cm.

The overall morphology of maize plants was visibly altered by light intensity and photoperiod (Figure 1). Plants grown under the P1600-H10 treatment displayed stronger growth, characterized by thicker stems and upright leaves, than those under low light (P800-H10, P800-H12). Under the 12 h d^−1^ photoperiod condition, especially at high light intensity (P1600-H12), plants showed relatively compact-type growth with smaller leaf angles compared with the 10 h d^−1^ photoperiod condition.

Stem length, stem diameter, leaf traits, and leaf angle further supported these observations (Table 1). Stem length did not differ significantly among treatments. Stem diameter was greater under higher light intensity, with the maximum value recorded in P1600-H10 as 19.8 mm. Leaf length decreased with increasing light intensity, the longest leaves were observed under P800-H12 (92.3 cm), while the shortest leaves occurred in P1600-H12 (83.2 cm). Leaf width increased slightly with light intensity under the 10 h d^−1^ photoperiod condition, reaching a maximum in P1600-H10 (10 cm), while under the 12 h d^−1^ photoperiod condition it did not differ significantly among treatments. Leaf angle was affected by both photoperiod and light intensity. In the same photoperiod, leaf angle decreased with increasing light intensity. Plants grown under a shorter photoperiod (10 h d^−1^) maintained wider leaf angles, whereas those exposed to a prolonged photoperiod (12 h d^−1^) showed more upward leaves. P1600-H12 exhibits the smallest leaf angle (15.8°).

### 2.2. Leaf Area and Biomass Accumulation of Maize Inbred Line

**Figure 2:**
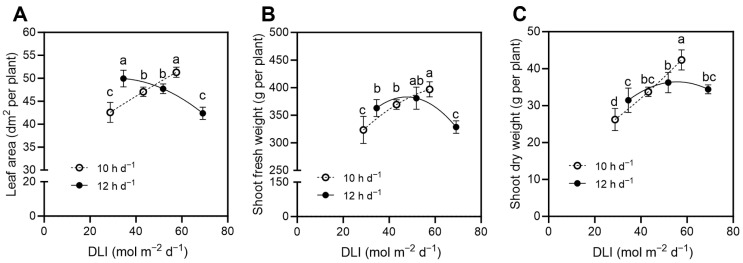
Leaf area (**A**), shoot fresh weight (**B**), and shoot dry weight (**C**) of maize inbred line (cv. Chang 7-2) in response to different DLI at V9 stage. Different letters for the same parameter indicate significant differences at the 5% level, according to Duncan’s multiple range test (n = 6).

Leaf area and biomass accumulation in the maize inbred line was affected by daily light integral (DLI) and displayed an increasing-then-decreasing trend across treatments (Figure 2). Leaf area exhibited distinct responses under different photoperiods (Figure 2A). Under the 10 h d^−1^ photoperiod condition, leaf area increased progressively with rising light intensity, reaching the maximum (51.3 dm^2^ per plant) at DLI = 57.6 mol m^−2^ d^−1^ (P1600-H10). In contrast, under the 12 h d^−1^ photoperiod condition, leaf area declined gradually as light intensity increased, with the highest value occurring at a moderate DLI of 34.6 mol m^−2^ d^−1^ (P800-H12). The maximum value of shoot fresh weight (396.9 g per plant) was observed at DLI = 57.6 mol m^−2^ d^−1^ (P1600-H10) (Figure 2B). Shoot dry weight followed a similar pattern (Figure 2C), reaching a maximum of 42.4 g per plant at DLI = 57.6 mol m^−2^ d^−1^ (P1600-H10), a value significantly higher than the other treatments. Collectively, these findings indicate that the P1600-H10 treatment (DLI = 57.6 mol m^−2^ d^−1^) achieved the most rapid vegetative growth, resulting in the greatest shoot fresh weight, shoot dry weight, and leaf area.

### 2.3. Photosynthetic Characteristics of Mazie Inbred Line

Photosynthetic characteristics varied significantly in response to changes in light intensity and photoperiod (Table 2). The net photosynthetic rate (P_n_) remained relatively stable across most treatments, ranging from 22.2 to 24.6 μmol m^−2^ s^−1^, but showed a significant decline under the highest DLI (P1600-H12; DLI = 69.1 mol m^−2^ d^−1^). This suggested that prolonged photoperiod combined with high light intensity led to photosynthetic inhibition, whereas moderate light levels under both photoperiods maintained higher P_n_ values. A slight decline in photosynthetic rate was observed under the lowest DLI treatment (P800-H10; DLI = 28.8 mol m^−2^ d^−1^), possibly due to insufficient light supply, which may have imposed constraints on early plant growth and development. Stomatal conductance (G_s_) followed a similar trend, with values between 0.064 and 0.074 mol m^−2^ s^−1^ in most treatments but dropping markedly in P1600-H12 (0.045 mol m^−2^ s^−1^). The high light exposure led to a reduction in intercellular CO_2_ concentration (C_i_), which decreased to 140 μmol mol^−1^ in P1600-H12, compared with 214–239 μmol mol^−1^ in the other treatments. Similarly, under the P1600-H12 treatment, the transpiration rate (T_r_) reached its minimum value (1.15 mmol m^−2^ s^−1^). These results suggest that the Higher light combined with longer day length (P1600-H12) led to stomatal limitation and reduced photosynthetic efficiency. The SPAD value, an indicator of chlorophyll content, was generally stable (41.9–45.5), with no consistent pattern among treatments.

**Figure 3:**
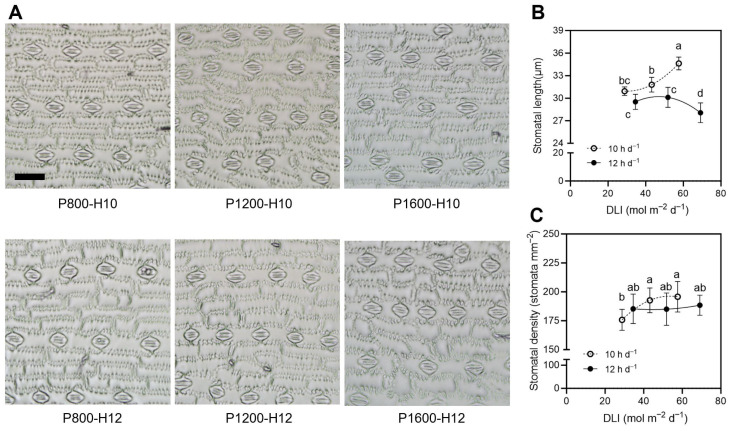
Effects of light intensity and photoperiod on stomatal traits of maize inbred line (cv. Chang 7-2) leaves at V9 stage. (**A**) Representative micrographs of stomata under different light treatments. Scale bar = 50 μm. (**B**) Stomatal length in response to DLI at different photoperiods. (**C**) Stomatal density in response to DLI at different photoperiods. Different letters indicate significant differences at the 5% level according to Duncan’s multiple range test (n = 6).

Stomatal traits were affected by light intensity and photoperiod (Figure 3). Under the 10 h d^−1^ photoperiod condition, stomatal length increased with rising light intensity, reaching its maximum (34.6 μm) at DLI = 57.6 mol m^−2^ d^−1^ (P1600-H10), which was significantly greater than that under low-DLI treatments. By contrast, under the 12 h d^−1^ photoperiod condition, stomatal length exhibited a unimodal trend, peaking at DLI = 51.9 mol m^−2^ d^−1^ (P1200-H12) and then declining markedly at the highest DLI of 69.1 mol m^−2^ d^−1^ (Figure 3B). Stomatal density varied less strongly (Figure 3C). Under the 10 h d^−1^ photoperiod condition, stomatal density tended to increase slightly with rising light intensity, reaching the highest values at a light intensity of 1600 μmol m^−2^ s^−1^. By comparison, under the 12 h d^−1^ photoperiod condition, no significant differences in stomatal density were observed among treatments, and the extent of variation remained smaller than that recorded for stomatal length. Collectively, these findings indicate that the combination of higher light intensity and a shorter photoperiod (P1600-H10) mainly enhanced stomatal function by promoting stomatal elongation rather than by increasing stomatal density. In contrast, the highest DLI (P1600-H12; DLI = 69.1 mol m^−2^ d^−1^) limited stomatal elongation, indicating that light intensity and photoperiod interactively affected leaf microstructure and, consequently, photosynthetic capacity.

### 2.4. Chlorophyll Fluorescence of Maize Inbred Line Leaves

**Figure 4:**
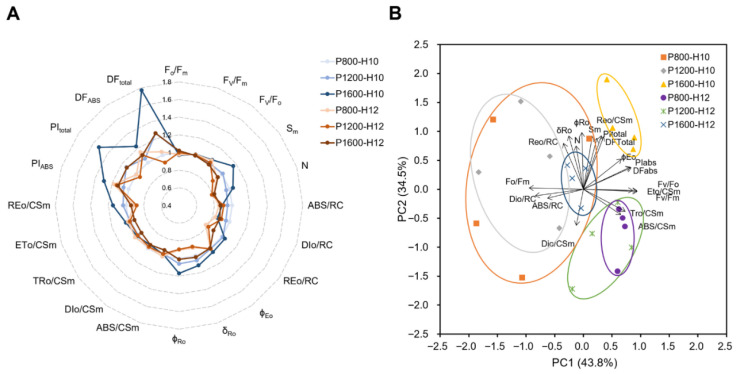
Effects of light intensity and photoperiod on chlorophyll fluorescence parameters derived from the JIP-test of maize inbred line (cv. Chang 7-2) leaves at V9 stage. (**A**) Radar plot showing normalized chlorophyll fluorescence parameters. (**B**) Principal component analysis of variability of selected JIP-test parameters.

Figure 4 shows how light intensity and photoperiod interactively affect chlorophyll fluorescence parameters in maize. The radar plot (Figure 4A) showed that P1600-H10 exhibited the strongest overall photochemical performance. This treatment displayed consistently higher values in JIP-test performance indices and driving-force parameters, including PI_ABS_ and PI_total_, indicating improved energy conservation from PSII light absorption to QB and PSI end-acceptor reduction, respectively, as well as higher DF_ABS_ and DF_total_, reflecting stronger photochemical driving forces at both the PSII level and across the overall electron transport chain (Table S1) [24,25]. By contrast, lower-light treatments (P800-H10 and P1200-H10) showed reduced PI values, lower φEO, and elevated DIo/CSm, indicating a shift toward non-photochemical quenching and limited photochemical utilization of absorbed light.

Principal component analysis of 19 chlorophyll fluorescence parameters was conducted under different light intensities (800, 1200, and 1600 μmol m^−2^ s^−1^) and photoperiods (10 and 12 h d^−1^). PC1 and PC2 explained 43.84% and 34.50% of total variation, respectively, giving a cumulative explanatory rate of 78.34% (Figure 4B). The six treatments were distinctly distributed in the PCA space defined by PC1 and PC2 (*p* = 0.002), indicating that different light regimes caused pronounced differences in the functional status of PSII. The P1600-H10 treatment was positioned at the positive end of PC1, reflecting the highest photosynthetic performance. It was strongly associated with high energy conversion efficiency and electron transport capacity, including PI_total_, PI_ABS_, DF_total_, and REo/RC. Under the 12 h d^−1^ photoperiod condition, treatments clustered near the origin and negative PC2 region. The negative PC2 axis was associated with low Sm and high DIo/CSm, suggesting increased thermal energy dissipation per unit leaf area and a reduced electron carrier pool on the PSII acceptor side under prolonged illumination.

### 2.5. Tassel Development of Maize Inbred Line at V9 Stage

**Figure 5:**
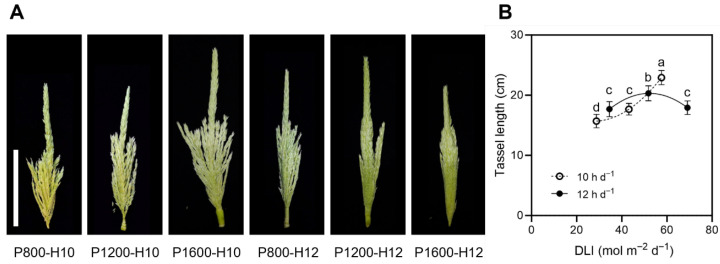
Effects of light intensity and photoperiod on tassel development of maize inbred line (cv. Chang 7-2) leaves at V9 stage. (**A**) Representative photographs of tassels under different light treatments. Scale bar = 10 cm. (**B**) Tassel length in response to DLI at different photoperiods. Different letters indicate significant differences at the 5% level according to Duncan’s multiple range test (n = 6).

Maize tassel morphology and length were significantly affected by light intensity and photoperiod (Figure 5A). Tassels developed more vigorously under higher light intensity with a 10 h d^−1^ photoperiod, whereas tassel growth was suppressed under the 12 h d^−1^ photoperiod condition. Tassel length (Figure 5B) revealed a nonlinear response to DLI. Under the 10 h d^−1^ photoperiod condition, tassel length increased progressively with increasing light intensity and reached a maximum of 22.9 cm at DLI = 57.6 mol m^−2^ d^−1^ (P1600-H10), significantly exceeding the values observed in all other treatments. In contrast, under the 12 h d^−1^ photoperiod condition, tassel length initially increased at moderate DLI but declined at the highest DLI (69.1 mol m^−2^ d^−1^), with the maximum length (20.3 cm) recorded at DLI = 51.9 mol m^−2^ d^−1^ (P1200-H12). These results indicate that the tassel development of maize was enhanced by high light intensity combined with a shorter photoperiod, with P1600-H10 providing the most favorable conditions for promoting tassel elongation. Excessive DLI or extended photoperiods suppressed tassel growth, suggesting that a balance between light intensity and photoperiod is crucial for tassel development.

## 3. Discussion

### 3.1. Effects of Light Intensity and Photoperiod on Growth and Photosystem Performance of Maize Inbred Line

Light intensity and photoperiod acted jointly to shape maize morphology, photosynthetic performance, and PSII photochemical behavior. Increasing light intensity from 800 to 1600 μmol m^−2^ s^−1^ consistently enhanced vegetative growth under the 10 h d^−1^ photoperiod condition, as reflected by greater stem diameter, higher biomass accumulation, expanded leaf area, and longer stomata (Table 1; Figure 2 and Figure 3). Under the 10 h d^−1^ photoperiod condition, light use efficiency and energy use efficiency remained relatively stable at high light intensities; no significant difference was detected between P1200-H10 and P1600-H10 (Figure S1). These responses are characteristic of high-light acclimation in C_4_ maize, where greater photon supply increases carbon assimilation and supports stronger biomass accumulation [26]. However, these positive effects depended strongly on photoperiod: under the 12 h d^−1^ photoperiod condition, the response of gas exchange to increasing light intensity became non-linear and, at high light intensity, inhibitory. Specifically, P_n_ declined under the P1600-H12 treatment relative to the optimal regime, and this decrease was accompanied by reductions in G_s_, C_i_ and stomatal length, indicating that stomatal limitation was a major constraint on photosynthesis under prolonged daily illumination (Table 2; Figure 3). In physiological terms, the concurrent decrease in G_s_ and C_i_ provides a diagnostic signature that the CO_2_ supply to the mesophyll became restricted, thereby lowering carboxylation substrate availability and suppressing net assimilation [27,28,29]. This interpretation is further strengthened when considering that maize is sensitive to photoperiod changes under controlled environments, where prolonged daylength can negatively affect developmental and physiological processes in certain genotypes [15]. Thus, in the present study, combining high light intensity with a longer photoperiod likely increased daily exposure to high irradiance to a level at which stomatal regulation shifted toward a protective mode, thereby constraining photosynthetic carbon gain.

Chlorophyll fluorescence results further demonstrated that high light combined with a moderate photoperiod (P1600-H10) most effectively supported PSII function. This treatment showed the highest PI_ABS_, PI_total_, φEo, REo/RC, and DF_total_, together with the lowest DIo/CSm, indicating efficient energy trapping and electron transport with minimal thermal dissipation. Principal component analysis also placed P1600-H10 distinctly along PC1, aligning with indicators of strong photochemical performance. In contrast, all 12 h d^−1^ photoperiod treatments clustered around the PC2 origin and negative axis, which were associated with higher energy dissipation and a reduced electron carrier pool. This demonstrates that prolonged daily exposure dampened PSII activity even when moderate light intensity was applied, consistent with previous findings that long photoperiods reduce photochemical efficiency in controlled environments [30,31].

Existing studies on light environment regulation in maize have mainly focused on shading and photoperiod effects [23,32,33], whereas our results suggested that maize performance during the V3-V9 stage is determined by the interaction between light intensity and photoperiod rather than by either factor alone. The superiority of the P1600-H10 treatment in biomass accumulation, stomatal traits, and PSII activity indicates that higher light intensity combined with an optimized photoperiod more effectively promoted maize growth. This response may be attributable to enhanced carbon assimilation and increased photosynthate accumulation, accompanied by the maintenance of stomatal regulation and photochemical efficiency.

### 3.2. Higher Light Intensity Combined with Optimized Photoperiod Enhances Tassel Development in Maize Inbred Line

The present findings have direct practical relevance for maize speed breeding under plant factory systems. Unlike conventional field conditions, plant factories allow precise manipulation of light intensity and photoperiod [34,35]. The results of this study demonstrate that maize responds most favorably to a high-intensity, moderate-photoperiod lighting strategy, rather than to prolonged illumination, highlighting the necessity of crop-specific light-environment design in plant factory systems [36]. The P1600-H10 lighting environment promoted the reproductive tassel development of the maize inbred line in the V9 stage. Based on tassel development at the V9 stage, the P1600-H10 treatment appears to provide a more favorable physiological condition for later tassel development in the maize inbred line. However, because flowering time and pollen quality were not directly measured in this study, its effects on reproductive timing and pollen performance require further verification [15]. Its ability to enhance growth rate, maintain PSII function, and support normal tassel development provides the physiological foundation for reducing the generation interval, particularly during early vegetative stages where biomass accumulation is rate-limiting. When integrated with early seed harvesting, embryo rescue, or rapid drying protocols [3,37], this lighting strategy could substantially shorten maize generation cycles in controlled environments.

Classical speed breeding protocols for wheat, barley, and other long-day crops rely heavily on extended photoperiods (20–22 h d^−1^) to accelerate developmental transitions [3,4,38]. However, maize sensitivity to long-day conditions—including delayed or blocked tassel initiation mediated by *ZmGI1* and *ZmGI2* pathways [18,19]—prevents direct application of these methods. The present results show that accelerated vegetative growth and stable reproductive development can be achieved under a shorter photoperiod, provided that light intensity is sufficiently high to drive rapid biomass accumulation. This aligns maize more closely with speed breeding strategies for photoperiod-sensitive species such as rice, pepper, hemp, and alfalfa, which rely on high light intensity, moderate photoperiod systems rather than photoperiod extension [6,8,10,39]. Speed breeding may involve trade-offs in yield-related traits, and a shorter generation cycle should not be directly translated to better reproductive performance. Previous studies in soybean and hemp have shown that light regimes designed to accelerate flowering or maturity may also alter plant architecture, pod number, seed yield, seed set, and overall vigor [9,10,40]. The P1600-H10 regime appears promising for maize speed breeding in promoting plant growth and improving tassel development, its effects on final grain yield and reproductive output still require further evaluation. Future studies should evaluate how light quality interacts with light intensity and photoperiod to regulate maize growth, reproductive development, and yield-related traits. Such research is essential for further optimizing light-environment strategies in maize speed breeding.

## 4. Materials and Methods

### 4.1. Plant Materials

The maize inbred line ‘Chang 7-2’ was used in this experiment. Seedlings were cultivated in an LED plant factory located at China Agricultural University (40°0′ N, 116°21′ E). Uniform and fully developed seeds were selected, thoroughly washed, and soaked in de-ionized water for 6 h. The seeds were then sown in 32-cell trays (W 280 mm × L 540 mm × H 50 mm) filled with a substrate consisting of 60% peat, 20% vermiculite, and 20% perlite (in volume). All areas designated for experimentation were evenly lit using white LED light (model W-LED-6000K, Beijing Lighting Valley Technology Co., Ltd., Beijing, China). The plants were grown under controlled environmental conditions with a light intensity of 600 μmol m^−2^ s^−1^ and a photoperiod of 16 h d^−1^. During the light period, the temperature was controlled at 27 ± 1 °C, relative humidity at 70 ± 10%, and CO_2_ concentration at 800 ± 50 μmol mol^−1^. During the dark period, the temperature was maintained at 21 ± 1 °C and relative humidity at 70 ± 10%, while CO_2_ concentration was not regulated. Eighteen days after sowing, the maize seedlings reach three fully expanded leaves (V3 stage, characterized by three fully expanded leaves and an average shoot fresh weight of approximately 4.8 g). Throughout the experimental period, all plants were supplied with Enshi nutrient solution (Japanese garden test formulation, in mg L^−1^): KNO_3_ 808.0, Ca(NO_3_)_2_ 944.0, MgSO_4_ 492.0, NH_4_H_2_PO_4_ 152.0, EDTA-Fe 30.0, H_3_BO_3_ 2.86, MnSO_4_·4H_2_O 2.13, ZnSO_4_·7H_2_O 0.22, CuSO_4_·5H_2_O 0.08, and (NH_4_)_6_Mo_7_O_24_·4H_2_O 0.02. The nutrient solution pH was maintained within a range of 5.5 to 6.0, and electrical conductivity was controlled between 2.0 and 2.4 mS cm^−1^. After seedling emergence, a regular fertigation regime was initiated, applying full-strength nutrient solution every three days.

### 4.2. Light Treatments

**Figure 6:**
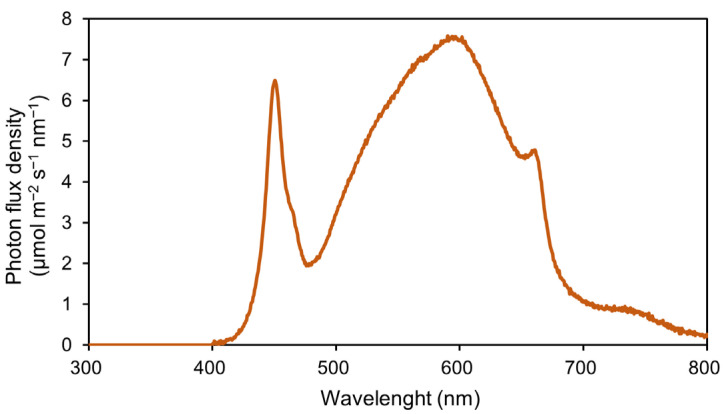
Spectral characteristics of the LED lighting environment in the experimental area.

Uniform and vigorous maize seedlings at the V3 stage were transplanted to 6 L pots (top diameter: 23 cm, height: 21.5 cm, bottom diameter: 18 cm). The pots were arranged in six cultivation beds, with 11 plants per bed. Each cultivation bed (0.72 m^2^) received one light treatment and was defined as the experimental unit for treatment application. Individual plants sampled from the same bed were regarded as one biological replicate. Six light treatments were established by combining three light intensities (800, 1200, and 1600 μmol m^−2^ s^−1^) with two photoperiods (10 h d^−1^ and 12 h d^−1^). These combinations resulted in DLIs ranging from 28.8 to 69.1 mol m^−2^ d^−1^ (Table 3). Treatments were designated as P800-H10, P1200-H10, P1600-H10, P800-H12, P1200-H12, and P1600-H12. For example, P800-H10 represents a light intensity of 800 μmol m^−2^ s^−1^ and photoperiod of 10 h d^−1^. Light was provided by LED lamps (model WR-LED5/1-200-P, Beijing Lighting Valley Technology Co., Ltd., Beijing, China) with a red: blue ratio of 1.8. The spectral distribution of the light in the range of 300–800 nm was measured 20 cm below the lamp using a fiber optic spectrometer (AvaSpec-ULS2048, Avantes, Apeldoorn, The Netherlands) (Figure 6). Light intensity was determined at a position 20 cm beneath the LED fixtures using a portable quantum sensor (LI-250A, LI-COR Inc., Lincoln, NE, USA). During the cultivation period, the temperature, relative humidity and CO_2_ concentration were maintained at 27 ± 1 °C, 70 ± 5%, and 800 μmol mol^−1^, respectively. During the dark period, the air temperature was adjusted to 21 ± 1 °C and the relative humidity was kept at 70 ± 5%, whereas CO_2_ levels were not controlled. All plants received Enshi nutrient solution prepared according to the method described in Section 2.1, and 200 mL of full-strength nutrient solution was supplied to each plant daily. The experiment ended 35 days after transplanting, when the maize plants had grown to nine fully expanded leaves (V9 stage).

### 4.3. Plant Morphological Measurements

At 35 days after transplanting—when maize plants reached the V9 stage—stem length, stem diameter, leaf length, leaf width, and leaf angle were measured. For morphological measurements, randomly selected plants from each cultivation bed were used as biological replicates. The number of sampled plants used for each parameter is indicated in the corresponding figure legends. The ninth fully expanded leaf was selected for measurements of leaf length, width, and leaf angle. Leaf angle was measured with a protractor, as the angle between the base of leaf blade and the stem. Stem diameter was measured at the base of each stem using a Vernier caliper. Shoots were harvested and shoot fresh weight was determined using an electronic balance (AX622ZH, Ohaus Instruments Co., Ltd., Shanghai, China). To measure leaf area, all leaves from each plant were flattened on a background panel alongside a reference marker (1 cm × 1 cm) and scanned using a scanner (CanoScan LiDE400; Canon Inc., Tokyo, Japan). The pixel areas of the leaves and the reference marker were quantified using Adobe Photoshop 2022 (Adobe Inc Co., Ltd., San Jose, CA, USA). Then the leaf area was calculated by normalizing the leaf pixel area against the marker (leaves pixel area/reference marker pixel area). Fresh shoot samples were placed in an oven at 105 °C for 3 h to terminate metabolic activity, after which they were oven-dried at 75 °C to a constant weight. After cooling to room temperature, the shoot dry weight was determined using the same balance. The morphology of the tassel was observed at the V9 stage of maize.

### 4.4. Photosynthetic Characteristics and Chlorophyll Fluorescence

At 35 days after transplanting, the ninth fully expanded leaf of each plant was selected for measurements. A portable photosynthesis system with a leaf chamber of 6400-02B (LI-6400XT, LI-COR Inc., Lincoln, NE, USA) was used to determine the net photosynthetic rate (P_n_), stomatal conductance (G_s_), intercellular CO_2_ concentration (C_i_), and transpiration rate (T_r_) during the light period. Measurement conditions were set as follows: light intensity of 1600 μmol m^−2^ s^−1^, air temperature of 27 °C, and CO_2_ concentration of 800 μmol mol^−1^. Photosynthetic parameters were determined at the whole-leaf level, using the ninth fully expanded leaf blade. For SPAD determination, six points were randomly selected from the middle portion of the ninth fully expanded leaf and measured using a SPAD meter (SPAD-502, Konica Minolta Inc., Tokyo, Japan). The average value was recorded as the SPAD value for each leaf. After 30 min of dark adaptation, the fast chlorophyll fluorescence induction kinetics (OJIP curves) were recorded using a multifunctional plant efficiency analyzer (M-PEA, Hansatech Instruments Ltd., King’s Lynn, UK). The JIP-test [26,41,42] was employed to analyze chlorophyll fluorescence transients. This test converts raw fluorescence data into a set of biophysical parameters that quantitatively describe energy fluxes through PSII. Principal component analysis was conducted based on JIP-test-derived parameters. For photosynthetic characteristics, SPAD determination, and chlorophyll fluorescence analysis, six plants per treatment were sampled, and each measured plant represented one biological replicate within the corresponding treatment.

Stomatal imprints of the abaxial epidermis were obtained during the light period using the nail-polish impression method. A uniform coating of clear nail polish was applied to the lower epidermal surface of the fully expanded ninth leaf. Once the polish had completely dried, the resulting film was carefully removed with forceps, placed onto a microscope slide, and sealed with a coverslip. Images were then captured under a light microscope (Olympus DP-72, Olympus Co., Ltd., Tokyo, Japan). Six plants were selected for each treatment as six biological replicates, and six fields of view were observed per leaf sample. Stomatal density was expressed as the number of stomata per unit leaf area (mm^−2^). In each field of view, six stomata were randomly selected, and their lengths (μm) were measured using ImageJ 1.54g software (National Institutes of Health, Bethesda, MD, USA).

### 4.5. Statistical Analyses

Data analysis and figure preparation were performed using SPSS 26 and GraphPad Prism 10, respectively. Differences among treatments were assessed using one-way ANOVA followed by Duncan’s multiple range test for mean separation at the 0.05 probability level. All results are presented as mean ± standard deviation (SD). Values followed by the same letter indicate no significant difference, whereas different letters denote statistically significant differences among treatments.

## 5. Conclusions

This study elucidated the effects of light intensity and photoperiod on morphology, photosynthetic performance, and stomatal and tassel development in the maize inbred line (cv. Chang 7-2). The results demonstrate that the light intensity of 1600 μmol m^−2^ s^−1^ and the 10 h d^−1^ photoperiod (DLI of 57.6 mol m^−2^ d^−1^) most effectively enhanced maize growth, photosynthetic capacity, and PSII performance while ensuring stable tassel development. This regime enhanced biomass production, stabilized tassel formation, and improved PSII performance, offering a practical and quantitative guideline for maize speed breeding systems under controlled environments.

## Figures and Tables

**Table 1:** Effects of light intensity and photoperiod on stem length, stem diameter, leaf morphology, and leaf angle of maize inbred line (cv. Chang 7-2) at V9 stage. Different letters within the same column indicate significant differences at the 5% level according to Duncan’s multiple range test (n = 6). “ns” indicates no significant difference.

Treatment	Stem Length (cm)		Stem Diameter (mm)		Leaf Length (cm)		Leaf Width (cm)		Leaf Angle (°)	
P800-H10	51.9 ± 3.4	ns	18.1 ± 0.9	b	91.9 ± 1.7	a	9.5 ± 0.3	b	20.0 ± 2.2	a
P1200-H10	52.8 ± 4.7	ns	19.0 ± 0.6	ab	91.6 ± 1.6	a	9.6 ± 0.2	ab	19.3 ± 1.4	a
P1600-H10	55.6 ± 2.9	ns	19.8 ± 0.2	a	88.1 ± 2.8	b	10.0 ± 0.2	a	18.5 ± 1.1	ab
P800-H12	56.2 ± 2.2	ns	18.6 ± 0.3	b	92.3 ± 1.1	a	9.7 ± 0.4	ab	17.8 ± 2.0	ab
P1200-H12	54.2 ± 2.8	ns	19.7 ± 0.4	a	88.1 ± 2.0	b	9.9 ± 0.3	ab	16.3 ± 1.5	b
P1600-H12	55.4 ± 4.1	ns	19.1 ± 0.7	ab	83.2 ± 2.3	c	9.7 ± 0.3	ab	15.8 ± 1.2	b

**Table 2:** Leaf gas-exchange parameters, including net photosynthetic rate (P_n_), stomatal conductance (G_s_), intercellular CO_2_ concentration (C_i_), and transpiration rate (T_r_), as well as SPAD values, of maize inbred line leaves at the V9 stage. Different letters within the same column indicate significant differences at the 5% level according to Duncan’s multiple range test (n = 5). “ns” indicates no significant difference.

Treatment	P_n_ (μmol m^−2^ s^−1^)		G_s_ (mol m^−2^ s^−1^)		C_i_ (μmol mol^−1^)		T_r_ (mmol m^−2^ s^−1^)		SPAD Value	
P800-H10	21.4 ± 2.5	b	0.064 ± 0.011	a	221 ± 26	a	1.45 ± 0.26	a	44.4 ± 3.3	ns
P1200-H10	23.0 ± 1.9	ab	0.070 ± 0.007	a	239 ± 20	a	1.56 ± 0.19	a	43.9 ± 4.1	ns
P1600-H10	22.2 ± 1.4	ab	0.068 ± 0.010	a	214 ± 27	a	1.36 ± 0.11	ab	45.5 ± 2.9	ns
P800-H12	23.5 ± 0.6	ab	0.070 ± 0.004	a	231 ± 15	a	1.50 ± 0.11	a	44.5 ± 4.1	ns
P1200-H12	24.6 ± 0.6	a	0.074 ± 0.008	a	232 ± 43	a	1.61 ± 0.14	a	45.3 ± 3.6	ns
P1600-H12	17.6 ± 2.4	c	0.045 ± 0.005	b	140 ± 20	b	1.15 ± 0.13	b	41.9 ± 2.1	ns

**Table 3:** Different light intensity and photoperiod treatments for maize inbred line plants.

Treatment	Light Intensity (μmol m^−2^ s^−1^)	Photoperiod (h d^−1^)	DLI (mol m^−2^ d^−1^)
P800-H10	800	10	28.8
P1200-H10	1200		43.2
P1600-H10	1600		57.6
P800-H12	800	12	34.6
P1200-H12	1200		51.9
P1600-H12	1600		69.1

## Data Availability

The original contributions presented in this study are included in the article/Supplementary Materials. Further inquiries can be directed to the corresponding author.

## Supplementary Materials

The following supporting information can be downloaded at: https://www.mdpi.com/article/10.3390/plants15081208/s1, Figure S1. Effects of light intensity and photoperiod on efficiency of capacity utilization of maize inbred line (cv. Chang 7-2); Table S1. Effects of light intensity and photoperiod on chlorophyll fluorescence parameters of maize inbred line (cv. Chang 7-2) leaves at the V9 stage; Table S2. Raw data of morphological indicators, corresponding to Table 1; Table S3. Raw data of leaf area and biomass accumulation, corresponding to Figure 2; Table S4. Raw data of photosynthetic characteristics, corresponding to Table 2; Table S5. Raw data of stomatal traits, corresponding to Figure 3; Table S6. Raw data of tassel length, corresponding to Figure 5; Table S7. Raw data of chlorophyll fluorescence, corresponding to Table S1.

## References

[1] He B., Pan S., Zhao J., Zou X., Liu X., Wu S. (2024). Maize Improvement Based on Modern Breeding Strategies: Progress and Perspective. ACS Agric. Sci. Technol..

[2] Xiong W., Reynolds M., Xu Y. (2022). Climate Change Challenges Plant Breeding. Curr. Opin. Plant Biol..

[3] Watson A., Ghosh S., Williams M.J., Cuddy W.S., Simmonds J., Rey M.-D., Asyraf Md Hatta M., Hinchliffe A., Steed A., Reynolds D. (2018). Speed Breeding Is a Powerful Tool to Accelerate Crop Research and Breeding. Nat. Plants.

[4] Hickey L.T., Hafeez A.N., Robinson H., Jackson S.A., Leal-Bertioli S.C.M., Tester M., Gao C., Godwin I.D., Hayes B.J., Wulff B.B.H. (2019). Breeding Crops to Feed 10 Billion. Nat. Biotechnol..

[5] Schoen A., Wallace S., Holbert M.F., Brown-Guidera G., Harrison S., Murphy P., Sanantonio N., Van Sanford D., Boyles R., Mergoum M. (2023). Reducing the Generation Time in Winter Wheat Cultivars Using Speed Breeding. Crop Sci..

[6] Han L., Lv Y., Zhang Y., Zhao X., Gao P., Liang Y., Li B. (2025). Optimizing the Light Intensity, Nutrient Solution, and Photoperiod for Speed Breeding of Alfalfa (Medicago sativa L.) Under Full-Spectrum LED Light. Agronomy.

[7] Wang G., Sun Z., Yang J., Ma Q., Wang X., Ke H., Huang X., Zhang L., Wang G., Gu Q. (2025). The Speed Breeding Technology of Five Generations per Year in Cotton. Theor. Appl. Genet..

[8] Liu Y., Li Z.-G., Cheng H., Yang X., Li M.-Y., Liu H.-Y., Gan R.-Y., Yang Q.-C. (2025). Plant Factory Speed Breeding Significantly Shortens Rice Generation Time and Enhances Metabolic Diversity. Engineering.

[9] Masangano M., Birhanie Z.M., Miao L., Wu L., Gao H., Wei P., Dong B., Koros D.K., Soltani M.Y., Mahamadou A.M. (2025). Impact of Light Quality on Accelerating Soybean Speed Breeding Efficiency Using LED-Based Systems. Discov. Plants.

[10] Schilling S., Melzer R., Dowling C.A., Shi J., Muldoon S., McCabe P.F. (2023). A Protocol for Rapid Generation Cycling (Speed Breeding) of Hemp (Cannabis sativa) for Research and Agriculture. Plant J..

[11] Samantara K., Bohra A., Mohapatra S.R., Prihatini R., Asibe F., Singh L., Reyes V.P., Tiwari A., Maurya A.K., Croser J.S. (2022). Breeding More Crops in Less Time: A Perspective on Speed Breeding. Biology.

[12] Sharma S., Kumar A., Dhakte P., Raturi G., Vishwakarma G., Barbadikar K.M., Das B.K., Shivaraj S.M., Sonah H., Deshmukh R. (2023). Speed Breeding Opportunities and Challenges for Crop Improvement. J. Plant Growth Regul..

[13] Aggarwal G., Jeena A.S., Mehra K., Kumar B., Kashyap S., Yadav D.K., Maurya A.K., Venkatesh S.C., Singla P., Bohra A. (2025). Speed-Bred Crops for Food Security and Sustainable Agriculture. Planta.

[14] Xu Y., Luo H., Zhang H., Yung W.-S., Li M.-W., Lam H.-M., Huang C. (2023). Feeding the World Using Speed Breeding Technology. Trends Plant Sci..

[15] Chen Q., Zhong H., Fan X., Li Y. (2015). An Insight into the Sensitivity of Maize to Photoperiod Changes under Controlled Conditions. Plant Cell Environ..

[16] Yang J., Wei H., Hou M., Chen L., Zou T., Ding H., Jing Y., Zhang X., Zhao Y., Liu Q. (2023). ZmSPL13 and ZmSPL29 Act Together to Promote Vegetative and Reproductive Transition in Maize. New Phytol..

[17] Singh I., Sheoran S., Kumar B., Kumar K., Rakshit S. (2021). Speed Breeding in Maize (Zea mays) Vis-à-Vis in Other Crops: Status and Prospects. Indian J. Agric. Sci..

[18] Bendix C., Mendoza J.M., Stanley D.N., Meeley R., Harmon F.G. (2013). The Circadian Clock-associated Gene Gigantea1 Affects Maize Developmental Transitions. Plant Cell Environ..

[19] Li Z., Gao F., Liu Y., Abou-Elwafa S.F., Qi J., Pan H., Hu X., Ren Z., Zeng H., Liu Z. (2023). ZmGI2 Regulates Flowering Time through Multiple Flower Development Pathways in Maize. Plant Sci..

[20] Miller T.A., Muslin E.H., Dorweiler J.E. (2008). A Maize CONSTANS-like Gene, Conz1, Exhibits Distinct Diurnal Expression Patterns in Varied Photoperiods. Planta.

[21] Lazakis C.M., Coneva V., Colasanti J. (2011). ZCN8 Encodes a Potential Orthologue of Arabidopsis FT Florigen That Integrates Both Endogenous and Photoperiod Flowering Signals in Maize. J. Exp. Bot..

[22] Wang J., Shi K., Lu W., Lu D. (2020). Post-Silking Shading Stress Affects Leaf Nitrogen Metabolism of Spring Maize in Southern China. Plants.

[23] Gao J., Liu Z., Zhao B., Dong S., Liu P., Zhang J. (2020). Shade Stress Decreased Maize Grain Yield, Dry Matter, and Nitrogen Accumulation. Agron. J..

[24] Goltsev V.N., Kalaji H.M., Paunov M., Bąba W., Horaczek T., Mojski J., Kociel H., Allakhverdiev S.I. (2016). Variable Chlorophyll Fluorescence and Its Use for Assessing Physiological Condition of Plant Photosynthetic Apparatus. Russ. J. Plant Physiol..

[25] Stirbet A., Govindjee (2011). On the Relation between the Kautsky Effect (Chlorophyll a Fluorescence Induction) and Photosystem II: Basics and Applications of the OJIP Fluorescence Transient. J. Photochem. Photobiol. B Biol..

[26] Franić M., Jambrović A., Šimić D., Galić V. (2019). Photosynthetic Properties of Maize Hybrids under Different Environmental Conditions Probed by the Chlorophyll a Fluorescence. Maydica.

[27] Sakoda K., Yamori W., Groszmann M., Evans J.R. (2021). Stomatal, Mesophyll Conductance, and Biochemical Limitations to Photosynthesis during Induction. Plant Physiol..

[28] Pilon C., Snider J.L., Sobolev V., Chastain D.R., Sorensen R.B., Meeks C.D., Massa A.N., Walk T., Singh B., Earl H.J. (2018). Assessing Stomatal and Non-Stomatal Limitations to Carbon Assimilation under Progressive Drought in Peanut (*Arachis hypogaea* L.). J. Plant Physiol..

[29] Elferjani R., Benomar L., Momayyezi M., Tognetti R., Niinemets Ü., Soolanayakanahally R.Y., Théroux-Rancourt G., Tosens T., Ripullone F., Bilodeau-Gauthier S. (2021). A Meta-Analysis of Mesophyll Conductance to CO_2_ in Relation to Major Abiotic Stresses in Poplar Species. J. Exp. Bot..

[30] Munyanont M., Lu N., Rachma D.F., Ruangsangaram T., Takagaki M. (2024). Lighting Patterns Regulate Flowering and Improve the Energy Use Efficiency of Calendula Cultivated in Plant Factories with Artificial Lighting. Agriculture.

[31] Gimeno T.E., Saavedra N., Ogée J., Medlyn B.E., Wingate L. (2019). A Novel Optimization Approach Incorporating Non-Stomatal Limitations Predicts Stomatal Behaviour in Species from Six Plant Functional Types. J. Exp. Bot..

[32] Yuan L., Liu J., Cai Z., Wang H., Fu J., Zhang H., Zhang Y., Zhu S., Wu W., Yan H. (2022). Shade Stress on Maize Seedlings Biomass Production and Photosynthetic Traits. Ciência Rural.

[33] Zhang K., Wei L., Geng J., Zhan W., Li Y., Shi Y., Zhang Y., Chen S., Yang J. (2025). Integrated Transcriptome and Metabolome Analysis Reveals the Impacts of Prolonged Light Exposure on Starch and Protein Content in Maize Kernels. BMC Genom..

[34] Aliniaeifard S., Azizi S., Zarbakhsh S., Esmaeili S., Baghalian K., Gruda N.S. (2025). Light in Controlled Environment Agriculture. Int. J. Veg. Sci..

[35] Liu X., Xu Y., Wang Y., Yang Q., Li Q. (2022). Rerouting Artificial Light for Efficient Crops Production: A Review of Lighting Strategy in PFALs. Agronomy.

[36] Sena S., Kumari S., Kumar V., Husen A. (2024). Light Emitting Diode (LED) Lights for the Improvement of Plant Performance and Production: A Comprehensive Review. Curr. Res. Biotechnol..

[37] Bhatta M., Sandro P., Smith M.R., Delaney O., Voss-Fels K.P., Gutierrez L., Hickey L.T. (2021). Need for Speed: Manipulating Plant Growth to Accelerate Breeding Cycles. Curr. Opin. Plant Biol..

[38] Li J., Zhang Y., Cheng R., Li T. (2025). Light Spectrum, Intensity, and Photoperiod Are Key for Production as Well as Speed Breeding of Spring Wheat in Indoor Farming. Plant Environ. Interact..

[39] Liu N., Ji F., Xu L., He D. (2019). Effects of LED Light Quality on the Growth of Pepper Seedling in Plant Factory. Int. J. Agric. Biol. Eng..

[40] Mitache M., Baidani A., Houasli C., Khouakhi K., Bencharki B., Idrissi O. (2023). Optimization of Light/Dark Cycle in an Extended Photoperiod-based Speed Breeding Protocol for Grain Legumes. Plant Breed..

[41] Strasserf R.J., Srivastava A., Govindjee (1995). Polyphasic Chlorophyll a Fluorescence Transient in Plants and Cyanobacteria. Photochem. Photobiol..

[42] Strasser R.J., Tsimilli-Michael M., Qiang S., Goltsev V. (2010). Simultaneous in Vivo Recording of Prompt and Delayed Fluorescence and 820-Nm Reflection Changes during Drying and after Rehydration of the Resurrection Plant Haberlea rhodopensis. Biochim. Biophys. Acta (BBA) Bioenerg..

