# Supplementary material for: Higher Light Intensity Combined with Optimized Photoperiod Enhances Growth and Tassel Development in Maize Inbred Line
Source: Plants (Basel). 2026 Apr 15;15(8):1208. doi: 10.3390/plants15081208 (PMC13120238; doi:10.3390/plants15081208)
Supplement: Supplementary file 1 [file plants-15-01208-s001.zip › plants-4217245-supplementary.pdf]

Supplementary Material

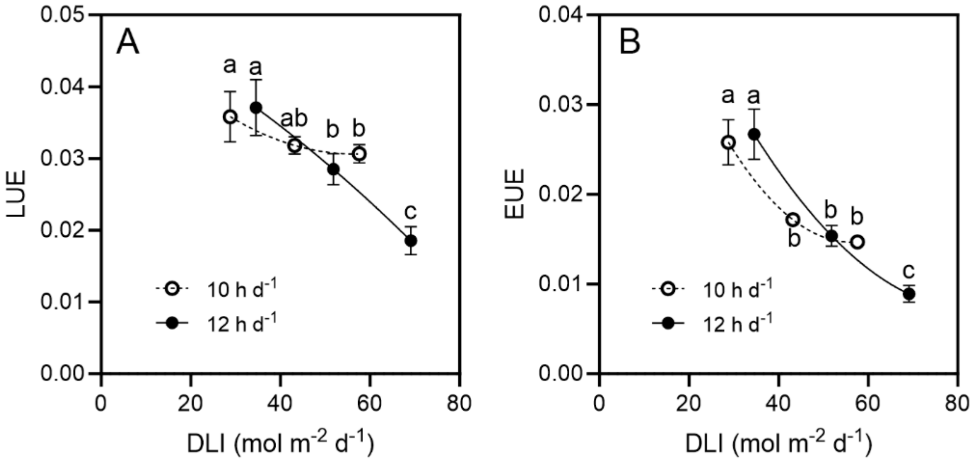

Figure S1. Effects of light intensity and photoperiod on efficiency of capacity utilization of maize inbred line (cv. Chang 7-2). (A) Light use efficiency (LUE), and (B) energy use efficiency (EUE) of maize inbred line (cv. Chang 7-2) in response to different DLI at V9 stage. Different letters for the same parameter indicate significant differences at the 5% level, according to Duncan's multiple range test (n = 5).

Table S1. Effects of light intensity and photoperiod on chlorophyll fluorescence parameters of maize inbred line (cv. Chang 7-2) leaves at the V9 stage. Different letters for the same parameter indicate significant differences at the 5% level, according to Duncan's multiple range test (n = 6). "ns" indicates no significant difference.

Treatment	PI_{ABS}	PI_{total}	DF_{ABS}	DF_{total}	F_v/F_m	S_m
P800-H10	3.25±0.45 b	1.81±0.42 b	0.50±0.06 b	0.24±0.10 b	0.78±0.01 ns	18.54±2.03 b
P1200-H10	3.44±0.31 b	2.06±0.29 b	0.53±0.04 b	0.31±0.06 b	0.78±0.01 ns	18.77±1.36 b
P1600-H10	4.20±0.31 a	2.76±0.43 a	0.62±0.03 a	0.44±0.06 a	0.79±0.01 ns	21.49±1.82 a
P800-H12	3.82±0.22 ab	1.80±0.13 b	0.58±0.02 ab	0.26±0.03 b	0.79±0.01 ns	17.22±0.95 bc
P1200-H12	3.71±0.69 ab	1.71±0.38 b	0.56±0.09 ab	0.22±0.09 b	0.78±0.01 ns	16.16±0.96 c
P1600-H12	3.65±0.38 ab	2.05±0.16 b	0.56±0.05 ab	0.31±0.03 b	0.77±0.01 ns	17.49±0.76 bc

Table S2. Raw data of morphological indicators, corresponding to Table 1.

Treatment	Rep	Stem length (cm)	Stem diameter (mm)	Leaf length (cm)	Leaf width (cm)	Leaf angle (°)
P800-H10	1	53.5	18.3	89	10.1	21
	2	45.5	16.7	92	9.2	17
	3	56.5	17	94	9.5	21
	4	51.5	18.5	94	9.5	22
	5	51	18.9	91	9.4	22
	6	53.5	18.9	91.5	9.5	17
P1200-H10	1	51.5	19.2	92.5	9.5	21
	2	50	18.8	89.5	9.8	20
	3	60	19	89.5	9.3	18
	4	45.5	19.2	92	9.7	20
	5	57	18	94	9.9	20
	6	53	20	92	9.6	17
P1600-H10	1	59	19.7	89.5	10.2	18
	2	60	20	82	9.6	20
	3	54.5	19.9	90	10.1	17
	4	54.5	20	89.5	9.9	18
	5	53.5	19.5	89	10.1	20
	6	52	19.7	88.5	9.8	18
P800-H12	1	58	18.7	92	10.3	19
	2	59	18.1	93	9.8	19
	3	52	19	93	9.5	16
	4	56	18.6	92	10	21
	5	56	18.4	93.5	9.5	15
	6	56	18.5	90	9.2	17
P1200-H12	1	57.5	19	90.5	9.5	18
	2	58.5	19.9	90	9.6	17
	3	52	19.5	85	9.7	14
	4	53.5	20.1	86	10.3	15
	5	52	19.8	89	10.2	18
	6	51.5	19.6	88	10	16
P1600-H12	1	58	19	84	9.3	17
	2	59.5	19.1	81	9.7	17
	3	49.5	18.6	87	9.7	15
	4	55.5	18.8	80	9.4	15
	5	50.5	18.3	83	10.2	17
	6	59.5	20.5	84	10	14

Table S3. Raw data of leaf area and biomass accumulation, corresponding to Figure 2.

Treatment	Rep	Leaf area (dm^2 per plant)	shoot fresh weight (g per plant)	shoot dry weight (g per plant)
P800-H10	1	40	296.27	21.31
	2	45.24	326.31	25.75
	3	45.12	298.78	26.2
	4	40.23	361.97	30.72
	5	43.65	347.35	28.92
	6	41.25	309.59	24.47
P1200-H10	1	49	375.99	33.25
	2	46.82	365.03	32.79
	3	46.53	369.32	33.54
	4	46.12	382.78	36.12
	5	47.7	354.41	34.55
	6	46.23	368.1	32.23
P1600-H10	1	53.09	414.91	43.53
	2	50.09	380.23	41.45
	3	52.08	383.72	37.52
	4	49.94	388.33	41.17
	5	51.65	402.13	45.85
	6	51.03	412.31	44.67
P800-H12	1	50.5	351.21	29.55
	2	53.2	353.53	29.52
	3	49.31	393.98	31.99
	4	47.12	349.31	38.54
	5	49.25	370.55	29.35
	6	50.25	360.02	29.78
P1200-H12	1	49.03	400.43	36.91
	2	46.05	358.38	32.92
	3	47.24	407.78	41.69
	4	48.99	353.83	34.68
	5	47.24	389	36.45
	6	47.74	376.46	34.91
P1600-H12	1	40.84	334.82	35.15
	2	42.91	332.84	35.08
	3	44.83	347.32	35.55
	4	41.23	321.17	35.04
	5	42.75	323.86	33.51
	6	41.69	311.01	32.17

Table S4. Raw data of photosynthetic characteristics, corresponding to Table 2.

Treatment	Rep	P_n (μmol m^{-2} s^{-1})	G_s (mol m^{-2} s^{-1})	C_i (μmol mol^{-1})	T_r (mmol m^{-2} s^{-1})	SPAD value
P800-H10	1	25.3	0.079	237	1.69	46.1
	2	22.9	0.0699	265	1.65	40.6
	3	17.8	0.051	195	1.07	40.2
	4	21.1	0.0703	201	1.63	48.1
	5	20.1	0.051	209	1.19	47
P1200-H10	1	20.4	0.058	211	1.73	47.1
	2	23.4	0.0759	266	1.65	49.8
	3	23.8	0.0712	220	1.23	40
	4	26	0.078	250	1.71	39
	5	21.6	0.0668	246	1.5	43.4
P1600-H10	1	22	0.0786	251	1.29	50.7
	2	21.2	0.0551	178	1.56	42
	3	20.4	0.0801	204	1.35	44.5
	4	23.4	0.059	198	1.22	44.7
	5	24.1	0.0692	241	1.4	45.8
P800-H12	1	23.7	0.0719	241	1.55	46.9
	2	24.1	0.0767	248	1.6	38.6
	3	22.7	0.0662	216	1.32	40.7
	4	24.1	0.0659	241	1.41	47.7
	5	23.1	0.0693	209	1.6	48.6
P1200-H12	1	25.4	0.088	293	1.74	48.3
	2	24.8	0.063	260	1.41	40.7
	3	24.9	0.075	182	1.71	50.5
	4	23.7	0.071	240	1.71	43.8
	5	24	0.0749	186	1.49	43.4
P1600-H12	1	19.9	0.0469	115	1.15	40.5
	2	13.5	0.036	175	0.92	44.4
	3	18.7	0.0482	141	1.28	44.4
	4	19.3	0.0499	133	1.24	40.5
	5	16.4	0.0449	137	1.18	39.5

Table S5. Raw data of stomatal traits, corresponding to Figure 3.

Treatment	Rep	Stomatal length (μm)	Stomatal density (stomata mm^{-2})
P800-H10	1	31.52	159.12
	2	30.67	173.04
	3	30.42	180.9
	4	30.1	187.99
	5	31.57	180.96
	6	31.3	172.88
P1200-H10	1	31.23	200.63
	2	31.99	176.88
	3	31.72	178.79
	4	32.45	198.55
	5	30.12	203.55
	6	33.26	197.63
P1600-H10	1	35.6	215.39
	2	35.63	203.71
	3	33.64	193.71
	4	34.05	180.79
	5	33.71	177.79
	6	35.1	202.88
P800-H12	1	28.96	175.96
	2	31.46	207.55
	3	28.72	172.96
	4	28.72	188.79
	5	29.06	172.96
	6	30.23	193.71
P1200-H12	1	30.26	180.79
	2	30.8	199.01
	3	28.33	178.88
	4	30.81	196.63
	5	32.02	195.55
	6	28.44	159.12
P1600-H12	1	28.59	178.96
	2	28.65	179.7
	3	25.52	186.79
	4	27.58	186.89
	5	28.21	193.71
	6	29.83	204.55

Table S6. Raw data of tassel length, corresponding to Figure 5.

Treatment	Rep	Tassel length (cm)
P800-H10	1	14.2
	2	16.8
	3	14.2
	4	16.9
	5	16.2
	6	15.8
P1200-H10	1	17.3
	2	17.6
	3	17.6
	4	16.1
	5	18
	6	19.4
P1600-H10	1	22
	2	21.1
	3	23.4
	4	22.9
	5	24.8
	6	23.3
P800-H12	1	18.8
	2	15.8
	3	18.45
	4	16.9
	5	19.3
	6	16.8
P1200-H12	1	21.5
	2	21.2
	3	18.3
	4	19.12
	5	21.56
	6	20.1
P1600-H12	1	19.2
	2	18
	3	16.2
	4	17.4
	5	17.2
	6	19.5

Table S7. Raw data of chlorophyll fluorescence, corresponding to Table S1.

Treatment	Rep	PI_{ABS}	PI_{total}	DF_{ABS}	DF_{total}	F_v/F_m	S_m
P800-H10	1	3.272	2.2406	0.5148	0.3504	0.77	20.0536
	2	3.774	1.975	0.5768	0.2956	0.8	18.7221
	3	3.371	1.6871	0.5208	0.2271	0.8	18.1445
	4	3.737	2.366	0.5625	0.3654	0.78	21.841
	5	2.657	1.1882	0.4244	0.0749	0.78	15.6477
	6	2.678	1.4228	0.4278	0.1561	0.77	16.8419
P1200-H10	1	3.606	1.9198	0.557	0.2833	0.79	17.827
	2	3.386	2.1673	0.5297	0.3359	0.78	18.4035
	3	3.391	2.456	0.5304	0.3902	0.78	20.6037
	4	3.291	1.6428	0.5174	0.2156	0.78	16.9391
	5	2.972	1.8181	0.4731	0.2596	0.77	18.3183
	6	3.998	2.3668	0.6018	0.3742	0.79	20.5489
P1600-H10	1	3.901	2.6509	0.5912	0.4234	0.79	20.338
	2	3.815	3.0031	0.5815	0.4776	0.77	22.9794
	3	4.079	2.3837	0.6105	0.3772	0.79	19.5992
	4	4.729	3.6006	0.6748	0.5564	0.79	24.7795
	5	4.253	2.5743	0.6287	0.4107	0.79	20.1802
	6	4.43	2.3365	0.6464	0.3686	0.78	21.0339
P800-H12	1	3.736	1.652	0.5724	0.218	0.79	16.9774
	2	3.44	1.804	0.5365	0.2588	0.79	17.529
	3	4.009	1.8697	0.603	0.2718	0.77	18.3368
	4	3.725	1.6082	0.5711	0.2063	0.79	15.2955
	5	3.899	1.8994	0.591	0.2786	0.79	17.5279
	6	4.122	1.986	0.6102	0.298	0.78	17.6833
P1200-H12	1	3.283	1.3141	0.5163	0.1186	0.78	14.8136
	2	2.416	1.2293	0.3831	0.0997	0.77	15.8848
	3	4.529	2.3025	0.656	0.3578	0.79	17.3673
	4	4.128	2.0368	0.6157	0.309	0.79	17.4324
	5	3.849	1.6843	0.5853	0.2264	0.78	16.0443
	6	4.056	1.7008	0.608	0.2319	0.79	15.4306
P1600-H12	1	3.969	2.3126	0.5986	0.3641	0.78	17.3157
	2	2.834	1.8733	0.4524	0.2726	0.76	17.9809
	3	3.952	1.968	0.5969	0.294	0.77	16.1993
	4	3.775	2.0863	0.577	0.3194	0.77	18.35
	5	3.741	1.8593	0.573	0.2693	0.78	16.9006
	6	3.655	2.1834	0.5629	0.3391	0.78	18.1875
